# Using Virtual Reality Exposure Therapy to Enhance Treatment of Anxiety Disorders: Identifying Areas of Clinical Adoption and Potential Obstacles

**DOI:** 10.3389/fpsyt.2019.00773

**Published:** 2019-10-25

**Authors:** Debra Boeldt, Elizabeth McMahon, Mimi McFaul, Walter Greenleaf

**Affiliations:** ^1^National Mental Health Innovation Center, University of Colorado Anschutz Medical Campus, Aurora, CO, United States; ^2^Independent Researcher, San Francisco, CA, United States; ^3^Department of Communication, Stanford University, Stanford, CA, United States

**Keywords:** virtual reality, anxiety, exposure therapy, virtual reality exposure therapy, technology, mental health

## Abstract

Despite strong evidence of effectiveness, exposure therapy is an underutilized treatment for anxiety disorders at a time when effective treatment for anxiety is greatly needed. The significant worldwide prevalence and negative impact of anxiety are documented and highlight the importance of increasing therapist and patient use of effective treatment. Obstacles to the use of exposure therapy are explored and steps to lessen these obstacles are proposed. In particular, virtual reality (VR) technology is discussed as a way to increase the availability of exposure therapy. Incorporating VR in therapy can increase the ease, acceptability, and effectiveness of treatment for anxiety. VR exposure therapy (VRET) permits individualized, gradual, controlled, immersive exposure that is easy for therapists to implement and often more acceptable to patients than *in vivo* or imaginal exposure. VR is presented as a scalable tool that can augment access to and effectiveness of exposure therapy thus improving treatment of anxiety disorders. VR also has the potential to help with assessment and with therapist training standardization. The authors advocate for providing continuing education in VRET to practicing clinicians and including training in exposure therapy and VRET in training programs. Ongoing development of VR applications for clinical use is encouraged, especially when developed in collaboration with software developers, clinical users, therapists who are experienced in VRET, and researchers.

## Introduction

Anxiety disorders are among the most common of mental disorders affecting nearly 18.1% of adults (1). The estimated prevalence of anxiety disorders worldwide is 7.3% (2), and they cause a high proportion of the global burden of disease (3). Anxiety symptoms can cause significant distress, impair quality of life, and increase stress. Anxiety increases risk for a range of co-occurring physical conditions, including chronic pain (4). Given the pervasiveness of anxiety and its impact on mental and physical health, effective treatment is clearly needed, yet a majority of affected individuals remain untreated (3, 5). These data highlight the importance for individuals and for society at large of increasing access to effective treatment. This perspective paper presents the ways that incorporating virtual reality (VR) in therapy can improve treatment for anxiety.

VR consists of a fully immersive, 3-D environment that transports people to engaging, interactive environments that can promote new learning. VR technology also has the potential to assist in training, evaluation, delivery, and supervision of psychotherapy skills (6), and can provide patients with a physiologically and emotionally evocative experience which can make VR a valuable tool for mental health treatment (7) VR exposure therapy (VRET) permits individualized, gradual, controlled, immersive exposure that is easy for therapists to implement and often more acceptable to patients than *in vivo* or imaginal exposure (8). This can allow users to practice behavioral skills in a safe environment through the support of a therapist. VRET has been used for the treatment of a range of conditions including social anxiety, Post-Traumatic Stress Disorder (PTSD), and panic disorder (9). The authors present several methods for incorporating VR technology into the therapeutic process and review how VRET can improve the ease, acceptability, and effectiveness of treatments for anxiety.

A patient can encounter difficulties finding or completing evidence-based anxiety treatment. These barriers can include failure to recognize and diagnose anxiety disorders, lack of access to treatment, and less-than-optimal quality of care (10). Since anxiety causes somatic symptoms, anxious patients often seek treatment through their primary care doctor (11). Anxiety disorders may go underrecognized and untreated. Access to effective care is further hampered by a shortage of therapists trained in evidence-based anxiety treatment (12). Limited access to effective treatment results in a large treatment gap allowing millions to suffer from anxiety even though evidence-based treatment exists (5). Obstacles to effective therapy need to be addressed.

Exposure therapy for anxiety disorders has a strong evidence base, yet few therapists utilize this method and patient and therapist misconceptions limit its availability (8, 13). Exposure therapy involves gradual and repeated exposure to feared stimuli with resultant changes in cognitions, behaviors, and emotional and physical responses. Feared stimuli can be almost anything: living organisms, inanimate objects, situations, activities, thoughts, mental images, physical symptoms, and/or affective experiences. Extensive research demonstrates the efficacy of exposure-based therapy for various anxiety disorders, especially phobias (14–16). Exposure facilitates extinction of the fear response and helps change dysfunctional assessments of threat and unhelpful responses, reducing the conditioned anxiety associated with feared stimuli (17, 18). Gradual exposure allows habituation and re-evaluation of the threat to occur.

Unfortunately, although exposure therapy can relieve anxiety symptoms ranging from mild to severe, therapists may not offer it and patients may be reluctant to try it, contributing to the treatment gap for anxiety (19). What are some obstacles to offering this proven effective treatment and how might VR help overcome these obstacles?

### Obstacles to Exposure Therapy

Several studies have explored why exposure therapy is offered less often than its proven effectiveness would warrant (20–23). Both patient and therapist concerns were uncovered. One barrier is patient fears of exposure therapy. Even when appropriately diagnosed and referred to a clinician offering exposure therapy, patients may refuse treatment or drop out if therapy requires prolonged exposure to their most feared stimuli.

A second barrier may be difficulties arranging exposure. The non-VR options for exposure are imaginal exposure (the patient confronts the feared stimulus in imagination) and *in vivo* exposure (the patient confronts the feared stimulus in actuality). Difficulties exist in both. In imaginal exposure, therapists cannot know or control what the patient imagines and the ability to create vivid mental images declines with age (24). *In vivo* exposure is often difficult or impossible to arrange inside the office and usually impractical to do outside the office. These above issues all interfere with making effective treatment for anxiety as widely available as is needed (20).

Therapist concerns about exposure therapy are a third barrier. Therapists often worry that exposure will be distressing for both therapist and patient and will increase patient drop out (8, 13, 21–23). When therapists have no way to control exposure, there is a risk that exposure may sensitize the patient and actually worsen anxiety. VR ameliorates this risk. Therapists can control different aspects of the patients’ experience during exposure, thus permitting gradual, repeatable, individualized exposure. This minimizes the risk of patient distress and maximizes the chance of patient success.

A fourth barrier is that relatively few mental health providers are trained in exposure therapy. For example, research supports imaginal exposure as a therapy for PTSD, yet a broad survey of community therapists regarding PTSD treatment found that the majority of the licensed psychologists in clinical practice were not using this evidence-based intervention (12). Given projections that in United States the current shortage of behavioral health providers will worsen by 2025, with approximately 40 million individuals not having access to behavioral healthcare (25), it is imperative that the available mental health providers offer the most effective treatments. How can this issue be addressed?

Formal clinical training is one way to correct therapist misconceptions of exposure treatment. Deacon et al. (26) reported that therapists who attended a day-long didactic workshop about exposure therapy showed a decrease in negative beliefs about the treatment approach and an increase in using it. Doctorate-level therapists report fewer reservations about exposure therapy compared to other mental health professionals, perhaps as a result of having more training opportunities (26). Disseminating evidence-based anxiety treatments is essential to ensure that the future mental health workforce has adequate training. Directly addressing clinicians’ concerns increases adoption as does training that incorporates motivational enhancement and/or offers a supportive learning community (22, 27). Even therapists trained and interested in exposure, such as imaginal exposure for PTSD, still underutilize the treatment (12, 28).

A final obstacle to offering exposure therapy may be time and/or difficulty involved, especially for *in vivo* exposure. *In vivo* exposure may be prohibited by clinic policy, can be difficult to arrange, take too long, and present confidentiality risks. These factors limit the availability of exposure therapy (28) despite its effectiveness. VR technology can help overcome these obstacles and support patient access to and acceptance of exposure therapy.

### Applications and Benefits of Virtual Reality

Motivation to integrate technology and behavioral health exists, as highlighted by the *All of Us* research initiative funded by the NIH (29). Presently available VR technology is being used to enhance treatment of anxiety and VR has the potential to improve clinical training (6). VR could create an engaging, controllable, repeatable, and safe training environment (30). Advances in VR allow users to enter a fully immersive environment and have simulated interactions with virtual humans (31). Practice with virtual humans has also demonstrated effectiveness as a way to train for difficult conversations (32). A recent study found that virtual patient simulations can be an effective tool for developing brief clinical interviewing skills among behavioral health providers (33).

In the near future, VR may help provide standardized clinical training in exposure therapy, making training easier and more accessible. A VR training environment would allow therapists to repeatedly practice with virtual patients while mastering clinical assessment and exposure therapy skills. VR might allow clinical supervisors to vary the scenarios and customize the virtual patients within the training environment. Practicing exposure therapy within VR could increase skills and decrease fears about delivering exposure therapy. Increased therapist comfort with and competence in exposure therapy will help therapists provide evidence-based treatment and counter patients’ fears about the therapy.

Researchers are also beginning to explore the potential of VR to help assess mental health conditions (34). VR-facilitated clinical assessment may improve the speed and accuracy of diagnosing patients’ anxiety disorders, allowing fast referral to appropriate treatment. Improved assessment and treatment should improve patient outcomes (35).

Clinically, VRET is a practical, empirically-based treatment that makes exposure therapy easier and more acceptable for therapists and patients (9). It can help patients learn and practice anxiety management skills and permits controlled, gradual exposure, which minimizes distress and optimizes treatment success. Practically, VR is increasingly affordable. The cost of VR software and hardware continues to decline, while the quantity and quality of VR content increases. VRET has been shown to reduce anxiety and phobia symptoms (36). Research on VRET has proliferated exploring its use with a range of mental health disorders, particularly anxiety disorders such as phobias (9). Over two decades of research exist documenting the effectiveness of VRET for anxiety disorders. Meta-analyses demonstrate a large effect size compared to a control or waitlist condition, and no significant difference in effect size or attrition rates when compared to the gold standard of *in vivo* exposure therapy (9, 36–39). To summarize, VRET is an acceptable and effective alternative to *in vivo* exposure.

VRET allows the therapist to see what the patient sees in the virtual environment. This addresses four limitations of imaginal exposure: not every patient imagines well; the ability to form mental images declines with age; the patient’s imagery may be too frightening; and the therapist neither knows nor controls what is being imagined. With VRET, the therapist can choose the VR content and personalize it for the patient. Therapists can guide patients through exposure in the office while monitoring and supporting the patient. Patients feel engaged, and the experience feels “real” but is a safe way for a patient to practice before facing a feared stimuli on their own in a real-world setting (34, 40). Therapists can monitor whether patients are attending to the content. Guiding patients to look at specific content and manage any anxiety that arises helps improve the efficiency of the treatment, ultimately improving patient outcomes. VRET also offers exposure therapy to patients who are resistant to other methods of exposure therapy (41). In the author’s (EM) clinical experience, VR has additional uses in psychotherapy beyond exposure. VR helps engage patients in treatment. Relaxing virtual environments (VEs) can help patients learn and practice anxiety management skills and can be used to reinforce patient involvement in treatment and increase positive therapeutic alliance.

The authors further propose that VRET might help manage clinic throughput and can lower costs associated with intensive anxiety treatment. VRET has demonstrated cost-effectiveness when compared to treatment as usual for PTSD (42). VRET offers the benefits of an *in vivo* experience in the office without requiring the burdensome time commitment required for therapists to guide, support, and monitor patients during real-world exposure. Such efficiency in providing effective, tailored exposure therapy can help therapists offer evidence-based treatment while handling the demands of a full caseload. Botella et al. (43) reported that successfully treating one phobia using VRET resulted in symptom relief of other, untreated, phobic behaviors. Effective treatment can be efficient treatment.

Lastly, this technology provides an opportunity to increase scalability. In addition to VR’s potential uses to improve therapist training, thus increasing the number of therapists with adequate training in exposure therapy. VR self-help interventions for people with distressing, but subclinical, anxiety may ease suffering and may prevent progression to more severe symptoms. A recent study investigated the efficacy of using a consumer, off-the-shelf VR hardware and software to conduct exposure therapy for fear of public speaking (44). The authors compared traditional therapist-led exposure to self-led exposure at home and found that improvements were comparable across both delivery methods. A single-blind, parallel-group randomized controlled trial showed that a low-cost, scalable intervention for fear of heights delivered by a virtual coach resulted in reduced symptoms (45).

### The Impact on Clinical Practice

The authors do not suggest that VRET will replace the need for trained therapists. The therapist’s clinical skills are the key factor to the effective use and implementation of VR (46). As research advances, people may use evidence-based VR self-help programs to prevent subclinical or mild to moderate anxiety from worsening, prepare for therapy when clinical anxiety is present, and use as an adjunct to therapy to get maximum benefit from every session thus saving patient, clinic, and therapist time. Such advances may help therapists manage their caseloads and allow them to focus limited resources on individuals with clinical anxiety. Treatments for specific phobia domains will also increase as has been demonstrated by an automated immersive therapy treatment for acrophobia (45). Such advances will help therapists maintain a manageable caseload and allow them to focus limited resources on individuals with clinical anxiety.

VR and VRET does have limitations. Virtual environments (VEs) are not commercially available for all anxiogenic stimuli. Cybersickness (nausea or discomfort in response to VR) can also limit VR’s use with some patients and limit the immersive effects of VR (47). Despite these limitations, VRET is an empirically supported treatment that makes exposure therapy easier and more acceptable. VR is increasingly affordable. The cost of VR software and hardware has declined, while the quantity and quality of VR content has increased.

### Additional Barriers to Adoption and Needs to Be Addressed

Several broader barriers exist to fully exploiting the potential of VR technology. First, the pace of new technology adoption by therapists is generally slow. Behavioral health interventions historically emphasize face-to-face delivery (29) relying on human judgment and assessment (6). This barrier may be reduced by increasing therapist knowledge of VRET’s research support, addressing therapist concerns, and offering training in VRET.

Second, therapists wishing to incorporate VR in treatment must decide which software and equipment best suits their needs and may struggle to find information about choosing and using VR. The growing number of VR products and apps can make it difficult to decide what to purchase. Administrators may worry about cost-effectiveness and benefit. This is likely to become less of a barrier as cost for VR equipment and products drops and as more patients request VRET. Publicizing the benefits of VRET to lay as well as professional audiences will increase patient demand, motivating therapists and administrators to offer VR.

Third, since new technology can affect delivery models and require more research and provider input in the process (48), ongoing research on the implementation of VR in a range of clinical settings is strongly recommended. Presently, not all patients become immersed in the currently available virtual environments (37). This may reflect differences in patient ability, therapist skills, and a need for more clinical VR content. Since patients present with complex problems, an expansive library of content is ideally needed for personalized, effective treatment. To date, research has primarily focused on applications of VR for phobias, social anxiety, obsessive–compulsive disorder, PTSD, eating disorders, psychosis, and substance use. Academic-industry partnerships are advancing the development of technology for mental health (32); however, inclusion criteria for studies is often restrictive. Input from a range of therapy providers in diverse clinical settings can help expand the use cases for VR.

Lastly, therapist interest in using VR in therapy is increasing, but training in VRET is generally unavailable. Normalizing exposure therapy approaches and minimizing clinician concerns about the safety and tolerability of exposure therapy are essential for successful dissemination (27). For technology to be effectively adopted, training needs to be available, clinically relevant, and focused on answering therapists’ concerns and needs. Resources should be developed that help therapists learn VRET and other therapeutic uses of VR. The authors advocate for the development of accredited continuing education classes and offering training in VRET to students training to become licensed mental health practitioners.

## Discussion

This paper reviews the need for evidence-based treatment of anxiety, the importance of training therapists in exposure therapy, the uses and benefits of VR technology to improve anxiety treatment and explores future applications of VR for training. VR may help reduce current obstacles to wider adoption of exposure therapy by making it more acceptable to patients and easier for therapists. VR is presented as a scalable tool that can augment access to and effectiveness of evidence-based exposure therapy.

In conclusion, research on VR applications and collaborative development of clinical content are recommended to move this tool forward in the field. Therapists experienced in VRET can serve as subject matter experts in the development of content. Training in VRET and other uses of VR should be offered in graduate school and through continuing education. Ongoing expansion of training in VRET for therapists is one way essential to address the vast need for anxiety treatment.

## Author Contributions

All authors listed have made a substantial, direct, and intellectual contribution to the work, and approved it for publication.

## Funding

The work of this group is grant-funded from Anschutz Foundation, Award #: N/A.

## Conflict of Interest

EM and WG are on the Scientific Advisory Board of Limbix, a company developing virtual reality content for mental health. NMHIC (DB and MM) provides consultation and has a partnership with Limbix.
